# 5-[(*tert*-Butyl­diphenyl­sil­yloxy)meth­yl]pyridazin-3(2*H*)-one

**DOI:** 10.1107/S160053681303167X

**Published:** 2013-11-27

**Authors:** María Carmen Costas-Lago, Tamara Costas, Noemí Vila, Carmen Terán

**Affiliations:** a Department of Organic Chemistry, University of Vigo, E-36310 Vigo, Spain

## Abstract

In the title compound, C_21_H_24_N_2_O_2_Si, a new pyridazin-3(2*H*)-one derivative, the carbonyl group of the heterocyclic ring and the O atom of the silyl ether are located on the same side of the pyridazinone ring and the C—C—O—Si torsion angle is −140.69 (17)°. In the crystal, mol­ecules are linked by pairs of strong N—H⋯O hydrogen bonds into centrosymmetric dimers with graph-set notation *R*_2_^2^(8). Weak C—H⋯π inter­actions are also observed.

## Related literature

For background to related compounds displaying biological activity, see: Siddiqui *et al.* (2010 ▶); Moos *et al.* (1987 ▶); Coelho *et al.* (2007 ▶); Abouzid & Bekhit (2008 ▶); Cesari *et al.* (2006 ▶); Rathish *et al.* (2009 ▶); Sivakumar *et al.* (2003 ▶); Al-Tel (2010 ▶); Suree *et al.* (2009 ▶); Tao *et al.* (2011 ▶); Weishaar *et al.* (1985 ▶). For related structures, see: Costas *et al.* (2010 ▶). For hydrogen-bond motifs, see: Bernstein *et al.* (1995 ▶).
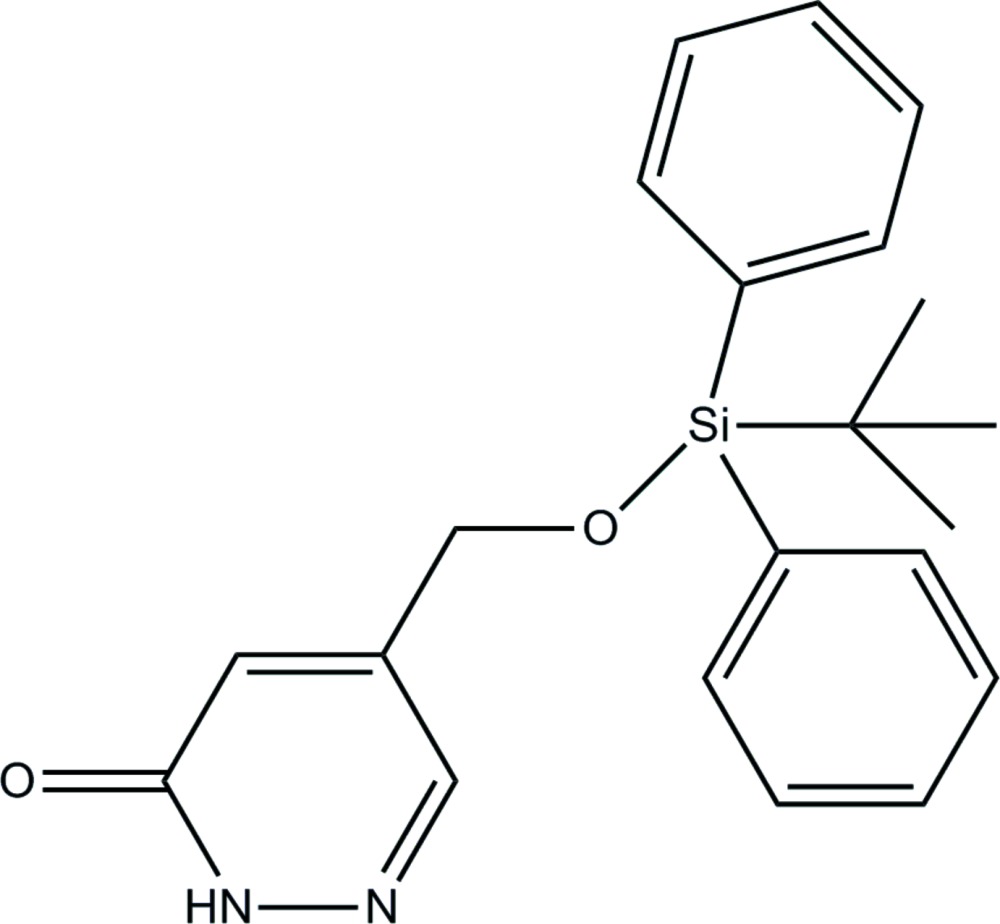


## Experimental

### 

#### Crystal data

C_21_H_24_N_2_O_2_Si
*M* *_r_* = 364.51
Monoclinic, 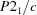

*a* = 7.9844 (10) Å
*b* = 14.1416 (17) Å
*c* = 18.553 (2) Å
β = 98.158 (2)°
*V* = 2073.6 (4) Å^3^
*Z* = 4
Mo *K*α radiation
μ = 0.13 mm^−1^
*T* = 293 K
0.49 × 0.47 × 0.35 mm

#### Data collection

Bruker SMART 1000 CCD diffractometer
Absorption correction: multi-scan (*SADABS*; Sheldrick, 1996 ▶) *T*_min_ = 0.704, *T*_max_ = 0.746
25441 measured reflections
5012 independent reflections
3090 reflections with *I* > 2σ(*I*)
*R*_int_ = 0.029

#### Refinement

*R*[*F* ^2^ > 2σ(*F* ^2^)] = 0.053
*wR*(*F* ^2^) = 0.172
*S* = 1.01
5012 reflections
242 parameters
H atoms treated by a mixture of independent and constrained refinement
Δρ_max_ = 0.39 e Å^−3^
Δρ_min_ = −0.21 e Å^−3^

### 

Data collection: *SMART* (Bruker, 1998 ▶); cell refinement: *SAINT* (Bruker, 1998 ▶); data reduction: *SAINT*; program(s) used to solve structure: *SIR2004* (Burla *et al.*, 2005 ▶); program(s) used to refine structure: *SHELXL97* (Sheldrick, 2008 ▶); molecular graphics: *PLATON* (Spek, 2003 ▶) and *Mercury* (Macrae *et al.*, 2006 ▶); software used to prepare material for publication: *SHELXTL* (Sheldrick, 2008 ▶).

## Supplementary Material

Crystal structure: contains datablock(s) I, New_Global_Publ_Block. DOI: 10.1107/S160053681303167X/bx2453sup1.cif

Structure factors: contains datablock(s) I. DOI: 10.1107/S160053681303167X/bx2453Isup2.hkl

Supplementary material file. DOI: 10.1107/S160053681303167X/bx2453Isup3.cml

Additional supplementary materials:  crystallographic information; 3D view; checkCIF report

## Figures and Tables

**Table 1:** Hydrogen-bond geometry (Å, °) *Cg*3 is the centroid of the C8′–C13′ ring.

*D*—H⋯*A*	*D*—H	H⋯*A*	*D*⋯*A*	*D*—H⋯*A*
N2—H2⋯O3^i^	0.89 (3)	1.93 (3)	2.812 (2)	173 (2)
C6—H6⋯*Cg*3^ii^	0.93	3.00	3.869 (3)	157

Symmetry codes: (i) 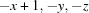; (ii) 
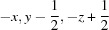
.

**Fig. 1.**
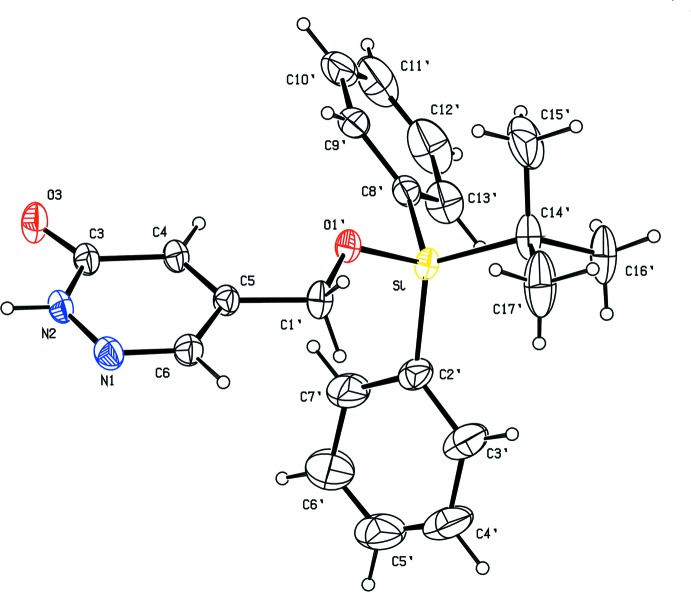
The molecular structure of (I) showing the atom-numbering scheme. Displacement ellipsoids are shown at the 20% probability level.

**Fig. 2.**
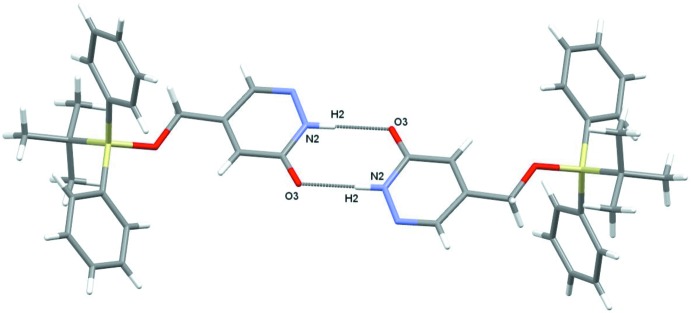
View of supramolecular dimer generated by NH···O hydrogen bonds.

## supplementary crystallographic information

### 1. Comment

Pyridazin-3(2*H*)-ones constitute an attractive building block for the designing and synthesis of new drugs. In many cases, the incorporation of a pyridazinone fragment in established biologically active molecules provides useful ligands for different targets. Thus, pyridazinone derivatives possess a wide variety of pharmacological properties, such as antihypertensive (Siddiqui *et al.*, 2010), cardiotonic (Moos *et al.*, 1987) and antiplatelet activities (Coelho *et al.*, 2007) and many of them have also been reported as anti-inflammatory (Abouzid & Bekhit, 2008), antinociceptive (Cesari *et al.*, 2006), antidiabetic (Rathish *et al.*, 2009), anticonvulsant (Sivakumar *et al.*, 2003), anticancer (Al-Tel, 2010), antimicrobial (Suree *et al.*, 2009) or anti-histamine H_3_ agents (Tao *et al.*, 2011). Most of pyridazinone derivatives previously described are 6-arylpyridazin-3(2*H*)-ones, a structure which was considered essential for cardiotonic and antiplatelet activities resulting from phosphodiesterase III inhibition (Weishaar *et al.*, 1985). However, the replacement of aryl by an alkyl chain functionalized with alcohol or ether groups gave rise to potent antiplatelet agents with a different mechanism of action (Costas *et al.*, 2010). In order to discover new pyridazinone analogues with this kind of activity, the titled compound I was synthesized and its crystal structure was determined.

The molecular structure of compound I, a new pyridazin-3(2*H*)-one derivative C5 substituted, is shows in figure 1. In the title compound the carbonyl group of the heterocyclic ring and the oxygen atom of the silyl ether are placed on the same side same side of the pyridazinone ring and the C5–C1'–O1'–Si torsion angle is -140.69 (17)°. The pyridazinone ring, a planar moiety, forms dihedral angles of 71.59 (10)° and 47.50 (10)°, respectively, with the C2'–C7' and C8'–C13' benzene rings, while the dihedral angle between both benzene rings is 73.07 (14)°. In the crystal structure the molecules are linked by N—H···O hydrogen bond interaction forming centrosymmetric ring with set-graph motif R_2_^2^(8), (Bernstein *et al.*, 1995),(Figure 2), Table 1. Weak C—H···π interactions are also observed

### 2. Experimental

A solution of 4-(*tert*-butyldiphenylsilyloxymethyl)-5-hydroxy-5*H*-furan-2-ona (50 mg, 0.136 mmol) and hydrazine monohydrate (14 ml, 0.284 mmol) in ethanol (2 ml) was stirred at reflux for 4 h. The solvent was evaporated under reduced pressure and residue was purified by column chromatography on silica gel (hexane/ethyl acetate 4:1) to afford a white solid (31 mg, 62%). Colourless block-like crystals suitable for X-ray analysis were obtained from a chloroform solution at room temperature.

### 3. Refinement

All H-atoms were positioned and refined using a riding model with d(C—H)= 0.93 Å, *U*_iso_ = 1.2*U*_eq_(C) for aromatic C—H groups,d(C—H)= 0.97 Å, *U*_iso_ = 1.2*U*_eq_(C) for CH_2_ group and d(C—H)= 0.96 Å, *U*_iso_ = 1.5*U*_eq_(C) for CH_3_ group; except for the hydrogen atoms of the NH group which were located from a Fourier-difference map and refined isotropically. The poor quality of the crystal, detected by its high mosaicity, explains the high value of the anisotropic displacement parameters corresponding to certain atoms, such as C4', C10', C11', C12', C17'. However, both data and model are good enough for a correct study.

### Crystal data

**Table:**

C_21_H_24_N_2_O_2_Si	*F*(000) = 776
*M**_r_* = 364.51	*D*_x_ = 1.168 Mg m^−^^3^
Monoclinic, *P*2_1_/*c*	Mo *K*α radiation, λ = 0.71073 Å
*a* = 7.9844 (10) Å	Cell parameters from 6082 reflections
*b* = 14.1416 (17) Å	θ = 2.2–25.6°
*c* = 18.553 (2) Å	µ = 0.13 mm^−^^1^
β = 98.158 (2)°	*T* = 293 K
*V* = 2073.6 (4) Å^3^	Prism, colourless
*Z* = 4	0.49 × 0.47 × 0.35 mm

### Data collection

**Table:**

Bruker SMART 1000 CCD diffractometer	5012 independent reflections
Radiation source: fine-focus sealed tube	3090 reflections with *I* > 2σ(*I*)
Graphite monochromator	*R*_int_ = 0.029
φ and ω scans	θ_max_ = 28.1°, θ_min_ = 1.8°
Absorption correction: multi-scan (*SADABS*; Sheldrick, 1996)	*h* = −10→10
*T*_min_ = 0.704, *T*_max_ = 0.746	*k* = −18→18
25441 measured reflections	*l* = −24→24

### Refinement

**Table:**

Refinement on *F*^2^	Primary atom site location: structure-invariant direct methods
Least-squares matrix: full	Secondary atom site location: difference Fourier map
*R*[*F*^2^ > 2σ(*F*^2^)] = 0.053	Hydrogen site location: inferred from neighbouring sites
*wR*(*F*^2^) = 0.172	H atoms treated by a mixture of independent and constrained refinement
*S* = 1.01	*w* = 1/[σ^2^(*F*_o_^2^) + (0.0697*P*)^2^ + 1.0269*P*] where *P* = (*F*_o_^2^ + 2*F*_c_^2^)/3
5012 reflections	(Δ/σ)_max_ < 0.001
242 parameters	Δρ_max_ = 0.39 e Å^−^^3^
0 restraints	Δρ_min_ = −0.21 e Å^−^^3^

### Special details

**Table:**

Experimental. ^1^H-RMN (400 MHz, CDCl_3_) *δ* p.p.m.: 12.91 (s, 1H), 7.72 (d, 1H, *J*=1.9 Hz), 7.67 (m, 4H), 7.44 (m, 6H), 7.08 (m, 1H), 4.61 (d, 2H, *J*=1.3 Hz), 1.12 (s, 9H).
Geometry. All e.s.d.'s (except the e.s.d. in the dihedral angle between two l.s. planes) are estimated using the full covariance matrix. The cell e.s.d.'s are taken into account individually in the estimation of e.s.d.'s in distances, angles and torsion angles; correlations between e.s.d.'s in cell parameters are only used when they are defined by crystal symmetry. An approximate (isotropic) treatment of cell e.s.d.'s is used for estimating e.s.d.'s involving l.s. planes.
Refinement. Refinement of *F*^2^ against ALL reflections. The weighted *R*-factor *wR* and goodness of fit *S* are based on *F*^2^, conventional *R*-factors *R* are based on *F*, with *F* set to zero for negative *F*^2^. The threshold expression of *F*^2^ > σ(*F*^2^) is used only for calculating *R*-factors(gt) *etc*. and is not relevant to the choice of reflections for refinement. *R*-factors based on *F*^2^ are statistically about twice as large as those based on *F*, and *R*- factors based on ALL data will be even larger.

### Fractional atomic coordinates and isotropic or equivalent isotropic displacement parameters (Å^2^)

**Table:**

	*x*	*y*	*z*	*U*_iso_*/*U*_eq_	
Si	−0.01100 (7)	0.06816 (5)	0.31744 (3)	0.0514 (2)	
N1	0.1582 (3)	−0.09069 (15)	0.02092 (11)	0.0662 (5)	
N2	0.2996 (3)	−0.03706 (14)	0.02689 (11)	0.0575 (5)	
H2	0.358 (3)	−0.0458 (19)	−0.0097 (15)	0.073 (8)*	
C4	0.2380 (3)	0.04087 (17)	0.13229 (11)	0.0544 (5)	
H4	0.2632	0.0852	0.1693	0.065*	
C3	0.3532 (3)	0.02690 (17)	0.08010 (11)	0.0545 (5)	
O3	0.4903 (2)	0.06809 (14)	0.08028 (9)	0.0743 (5)	
C5	0.0949 (3)	−0.00962 (16)	0.12787 (11)	0.0523 (5)	
C6	0.0614 (3)	−0.07715 (17)	0.07036 (13)	0.0616 (6)	
H6	−0.0361	−0.1137	0.0683	0.074*	
C1'	−0.0338 (3)	0.0012 (2)	0.17870 (12)	0.0650 (6)	
H1'1	−0.1425	0.0169	0.1510	0.078*	
H1'2	−0.0458	−0.0584	0.2034	0.078*	
O1'	0.0132 (2)	0.07213 (11)	0.23069 (8)	0.0597 (4)	
C2'	0.0633 (4)	−0.05142 (19)	0.35288 (13)	0.0709 (7)	
C3'	−0.0335 (6)	−0.1239 (3)	0.37389 (18)	0.1092 (12)	
H3'	−0.1494	−0.1151	0.3724	0.131*	
C4'	0.0403 (9)	−0.2126 (3)	0.3980 (2)	0.1304 (18)	
H4'	−0.0254	−0.2612	0.4129	0.156*	
C5'	0.2058 (10)	−0.2236 (3)	0.3983 (2)	0.145 (2)	
H5'	0.2548	−0.2811	0.4138	0.174*	
C6'	0.3032 (8)	−0.1568 (4)	0.3776 (3)	0.165 (2)	
H6'	0.4180	−0.1676	0.3775	0.198*	
C7'	0.2323 (5)	−0.0705 (3)	0.3562 (3)	0.1212 (14)	
H7'	0.3028	−0.0229	0.3434	0.145*	
C8'	0.1407 (3)	0.15851 (17)	0.35988 (14)	0.0613 (6)	
C9'	0.2043 (4)	0.22855 (19)	0.3190 (2)	0.0853 (9)	
H9'	0.1707	0.2305	0.2689	0.102*	
C10'	0.3164 (5)	0.2953 (3)	0.3512 (4)	0.1315 (19)	
H10'	0.3586	0.3412	0.3227	0.158*	
C11'	0.3645 (5)	0.2945 (4)	0.4228 (4)	0.156 (3)	
H11'	0.4399	0.3401	0.4439	0.187*	
C12'	0.3054 (5)	0.2284 (4)	0.4651 (3)	0.1347 (18)	
H12'	0.3393	0.2289	0.5151	0.162*	
C13'	0.1941 (4)	0.1597 (3)	0.43397 (17)	0.0907 (9)	
H13'	0.1548	0.1138	0.4634	0.109*	
C14'	−0.2325 (3)	0.1025 (3)	0.32926 (15)	0.0812 (9)	
C15'	−0.2574 (5)	0.2050 (4)	0.3026 (2)	0.1399 (18)	
H15A	−0.3708	0.2249	0.3063	0.210*	
H15B	−0.2382	0.2089	0.2527	0.210*	
H15C	−0.1787	0.2454	0.3320	0.210*	
C16'	−0.2605 (5)	0.0971 (4)	0.40913 (18)	0.1222 (15)	
H16A	−0.3718	0.1197	0.4137	0.183*	
H16B	−0.1777	0.1355	0.4383	0.183*	
H16C	−0.2494	0.0327	0.4255	0.183*	
C17'	−0.3641 (4)	0.0404 (4)	0.2832 (2)	0.1413 (19)	
H17A	−0.3467	−0.0245	0.2974	0.212*	
H17B	−0.3524	0.0472	0.2327	0.212*	
H17C	−0.4757	0.0597	0.2907	0.212*	

### Atomic displacement parameters (Å^2^)

**Table:**

	*U*^11^	*U*^22^	*U*^33^	*U*^12^	*U*^13^	*U*^23^
Si	0.0496 (3)	0.0634 (4)	0.0439 (3)	−0.0037 (3)	0.0161 (2)	0.0005 (3)
N1	0.0711 (13)	0.0649 (13)	0.0656 (12)	0.0004 (10)	0.0206 (10)	−0.0141 (10)
N2	0.0594 (11)	0.0651 (12)	0.0513 (10)	0.0080 (9)	0.0197 (9)	−0.0085 (9)
C4	0.0579 (13)	0.0641 (14)	0.0436 (11)	0.0038 (10)	0.0150 (9)	−0.0058 (10)
C3	0.0551 (13)	0.0638 (14)	0.0470 (11)	0.0066 (11)	0.0154 (9)	−0.0025 (10)
O3	0.0665 (11)	0.0986 (14)	0.0636 (10)	−0.0144 (10)	0.0295 (8)	−0.0208 (9)
C5	0.0554 (12)	0.0599 (13)	0.0437 (11)	0.0069 (10)	0.0138 (9)	0.0040 (9)
C6	0.0643 (14)	0.0616 (14)	0.0613 (14)	−0.0034 (11)	0.0173 (11)	−0.0043 (11)
C1'	0.0585 (13)	0.0889 (18)	0.0508 (12)	−0.0025 (12)	0.0192 (10)	−0.0059 (12)
O1'	0.0700 (10)	0.0678 (10)	0.0463 (8)	0.0021 (8)	0.0256 (7)	0.0008 (7)
C2'	0.100 (2)	0.0637 (15)	0.0507 (13)	−0.0123 (14)	0.0163 (13)	0.0017 (11)
C3'	0.158 (3)	0.084 (2)	0.089 (2)	−0.042 (2)	0.026 (2)	0.0057 (18)
C4'	0.236 (6)	0.075 (3)	0.086 (2)	−0.041 (3)	0.041 (3)	0.0092 (19)
C5'	0.257 (7)	0.086 (3)	0.098 (3)	0.015 (4)	0.048 (4)	0.023 (2)
C6'	0.170 (5)	0.123 (4)	0.210 (6)	0.064 (4)	0.057 (4)	0.072 (4)
C7'	0.110 (3)	0.086 (2)	0.173 (4)	0.028 (2)	0.037 (3)	0.051 (2)
C8'	0.0532 (12)	0.0578 (14)	0.0755 (16)	0.0026 (11)	0.0181 (11)	−0.0091 (12)
C9'	0.0749 (18)	0.0550 (15)	0.135 (3)	0.0015 (13)	0.0461 (18)	−0.0044 (16)
C10'	0.098 (3)	0.062 (2)	0.253 (6)	−0.0135 (19)	0.087 (4)	−0.037 (3)
C11'	0.074 (3)	0.129 (4)	0.273 (8)	−0.034 (2)	0.056 (4)	−0.111 (5)
C12'	0.085 (3)	0.169 (4)	0.144 (4)	−0.006 (3)	−0.006 (2)	−0.085 (3)
C13'	0.0783 (19)	0.108 (2)	0.083 (2)	−0.0066 (17)	0.0006 (15)	−0.0245 (18)
C14'	0.0536 (14)	0.131 (3)	0.0633 (15)	0.0030 (16)	0.0236 (12)	−0.0113 (17)
C15'	0.096 (3)	0.180 (5)	0.152 (4)	0.074 (3)	0.042 (2)	0.021 (3)
C16'	0.092 (2)	0.209 (4)	0.075 (2)	−0.002 (3)	0.0472 (18)	−0.029 (2)
C17'	0.0533 (17)	0.266 (6)	0.107 (3)	−0.023 (3)	0.0197 (17)	−0.057 (3)

### Geometric parameters (Å, º)

**Table:**

Si—O1'	1.6490 (15)	C6'—C7'	1.380 (5)
Si—C8'	1.857 (3)	C6'—H6'	0.9300
Si—C14'	1.877 (3)	C7'—H7'	0.9300
Si—C2'	1.880 (3)	C8'—C13'	1.380 (4)
N1—C6	1.295 (3)	C8'—C9'	1.387 (4)
N1—N2	1.351 (3)	C9'—C10'	1.378 (5)
N2—C3	1.362 (3)	C9'—H9'	0.9300
N2—H2	0.89 (3)	C10'—C11'	1.329 (8)
C4—C5	1.340 (3)	C10'—H10'	0.9300
C4—C3	1.440 (3)	C11'—C12'	1.348 (8)
C4—H4	0.9300	C11'—H11'	0.9300
C3—O3	1.240 (3)	C12'—C13'	1.386 (5)
C5—C6	1.429 (3)	C12'—H12'	0.9300
C5—C1'	1.498 (3)	C13'—H13'	0.9300
C6—H6	0.9300	C14'—C16'	1.531 (4)
C1'—O1'	1.406 (3)	C14'—C17'	1.533 (5)
C1'—H1'1	0.9700	C14'—C15'	1.535 (5)
C1'—H1'2	0.9700	C15'—H15A	0.9600
C2'—C7'	1.369 (5)	C15'—H15B	0.9600
C2'—C3'	1.373 (4)	C15'—H15C	0.9600
C3'—C4'	1.431 (6)	C16'—H16A	0.9600
C3'—H3'	0.9300	C16'—H16B	0.9600
C4'—C5'	1.329 (7)	C16'—H16C	0.9600
C4'—H4'	0.9300	C17'—H17A	0.9600
C5'—C6'	1.315 (7)	C17'—H17B	0.9600
C5'—H5'	0.9300	C17'—H17C	0.9600
			
O1'—Si—C8'	103.33 (10)	C2'—C7'—H7'	118.4
O1'—Si—C14'	110.38 (11)	C6'—C7'—H7'	118.4
C8'—Si—C14'	109.94 (13)	C13'—C8'—C9'	116.8 (3)
O1'—Si—C2'	107.29 (10)	C13'—C8'—Si	121.4 (2)
C8'—Si—C2'	108.43 (12)	C9'—C8'—Si	121.8 (2)
C14'—Si—C2'	116.61 (15)	C10'—C9'—C8'	121.1 (4)
C6—N1—N2	115.8 (2)	C10'—C9'—H9'	119.4
N1—N2—C3	127.22 (19)	C8'—C9'—H9'	119.4
N1—N2—H2	112.7 (17)	C11'—C10'—C9'	120.4 (5)
C3—N2—H2	120.1 (17)	C11'—C10'—H10'	119.8
C5—C4—C3	120.4 (2)	C9'—C10'—H10'	119.8
C5—C4—H4	119.8	C10'—C11'—C12'	120.8 (4)
C3—C4—H4	119.8	C10'—C11'—H11'	119.6
O3—C3—N2	120.03 (19)	C12'—C11'—H11'	119.6
O3—C3—C4	125.6 (2)	C11'—C12'—C13'	120.1 (5)
N2—C3—C4	114.4 (2)	C11'—C12'—H12'	120.0
C4—C5—C6	118.0 (2)	C13'—C12'—H12'	120.0
C4—C5—C1'	124.2 (2)	C8'—C13'—C12'	120.8 (4)
C6—C5—C1'	117.7 (2)	C8'—C13'—H13'	119.6
N1—C6—C5	124.1 (2)	C12'—C13'—H13'	119.6
N1—C6—H6	118.0	C16'—C14'—C17'	109.2 (3)
C5—C6—H6	118.0	C16'—C14'—C15'	109.2 (3)
O1'—C1'—C5	111.4 (2)	C17'—C14'—C15'	108.3 (3)
O1'—C1'—H1'1	109.4	C16'—C14'—Si	111.7 (2)
C5—C1'—H1'1	109.4	C17'—C14'—Si	111.7 (2)
O1'—C1'—H1'2	109.4	C15'—C14'—Si	106.7 (2)
C5—C1'—H1'2	109.4	C14'—C15'—H15A	109.5
H1'1—C1'—H1'2	108.0	C14'—C15'—H15B	109.5
C1'—O1'—Si	126.05 (15)	H15A—C15'—H15B	109.5
C7'—C2'—C3'	115.6 (3)	C14'—C15'—H15C	109.5
C7'—C2'—Si	116.9 (2)	H15A—C15'—H15C	109.5
C3'—C2'—Si	127.5 (3)	H15B—C15'—H15C	109.5
C2'—C3'—C4'	121.3 (4)	C14'—C16'—H16A	109.5
C2'—C3'—H3'	119.3	C14'—C16'—H16B	109.5
C4'—C3'—H3'	119.3	H16A—C16'—H16B	109.5
C5'—C4'—C3'	118.0 (4)	C14'—C16'—H16C	109.5
C5'—C4'—H4'	121.0	H16A—C16'—H16C	109.5
C3'—C4'—H4'	121.0	H16B—C16'—H16C	109.5
C6'—C5'—C4'	122.9 (5)	C14'—C17'—H17A	109.5
C6'—C5'—H5'	118.6	C14'—C17'—H17B	109.5
C4'—C5'—H5'	118.6	H17A—C17'—H17B	109.5
C5'—C6'—C7'	119.0 (5)	C14'—C17'—H17C	109.5
C5'—C6'—H6'	120.5	H17A—C17'—H17C	109.5
C7'—C6'—H6'	120.5	H17B—C17'—H17C	109.5
C2'—C7'—C6'	123.3 (4)		
			
C6—N1—N2—C3	2.2 (4)	C3'—C2'—C7'—C6'	1.3 (6)
N1—N2—C3—O3	176.7 (2)	Si—C2'—C7'—C6'	−176.7 (4)
N1—N2—C3—C4	−3.9 (3)	C5'—C6'—C7'—C2'	−2.3 (9)
C5—C4—C3—O3	−178.2 (2)	O1'—Si—C8'—C13'	−161.8 (2)
C5—C4—C3—N2	2.5 (3)	C14'—Si—C8'—C13'	80.3 (2)
C3—C4—C5—C6	0.2 (3)	C2'—Si—C8'—C13'	−48.2 (2)
C3—C4—C5—C1'	−178.8 (2)	O1'—Si—C8'—C9'	18.9 (2)
N2—N1—C6—C5	1.0 (4)	C14'—Si—C8'—C9'	−98.9 (2)
C4—C5—C6—N1	−2.1 (4)	C2'—Si—C8'—C9'	132.6 (2)
C1'—C5—C6—N1	177.0 (2)	C13'—C8'—C9'—C10'	0.5 (4)
C4—C5—C1'—O1'	1.1 (3)	Si—C8'—C9'—C10'	179.8 (2)
C6—C5—C1'—O1'	−177.9 (2)	C8'—C9'—C10'—C11'	−0.8 (5)
C5—C1'—O1'—Si	−140.69 (17)	C9'—C10'—C11'—C12'	0.2 (7)
C8'—Si—O1'—C1'	160.37 (19)	C10'—C11'—C12'—C13'	0.5 (7)
C14'—Si—O1'—C1'	−82.1 (2)	C9'—C8'—C13'—C12'	0.3 (4)
C2'—Si—O1'—C1'	45.9 (2)	Si—C8'—C13'—C12'	−179.0 (3)
O1'—Si—C2'—C7'	66.8 (3)	C11'—C12'—C13'—C8'	−0.8 (6)
C8'—Si—C2'—C7'	−44.2 (3)	O1'—Si—C14'—C16'	178.5 (3)
C14'—Si—C2'—C7'	−168.9 (3)	C8'—Si—C14'—C16'	−68.1 (3)
O1'—Si—C2'—C3'	−110.9 (3)	C2'—Si—C14'—C16'	55.8 (3)
C8'—Si—C2'—C3'	138.1 (3)	O1'—Si—C14'—C17'	56.0 (3)
C14'—Si—C2'—C3'	13.4 (3)	C8'—Si—C14'—C17'	169.4 (3)
C7'—C2'—C3'—C4'	0.4 (5)	C2'—Si—C14'—C17'	−66.7 (3)
Si—C2'—C3'—C4'	178.2 (3)	O1'—Si—C14'—C15'	−62.1 (3)
C2'—C3'—C4'—C5'	−1.1 (6)	C8'—Si—C14'—C15'	51.2 (3)
C3'—C4'—C5'—C6'	0.1 (8)	C2'—Si—C14'—C15'	175.1 (2)
C4'—C5'—C6'—C7'	1.6 (9)		

### Hydrogen-bond geometry (Å, º)

Cg3 is the centroid of the C8'–C13' ring.

**Table:**

*D*—H···*A*	*D*—H	H···*A*	*D*···*A*	*D*—H···*A*
N2—H2···O3^i^	0.89 (3)	1.93 (3)	2.812 (2)	173 (2)
C6—H6···*Cg*3^ii^	0.93	3.00	3.869 (3)	157

Symmetry codes: (i) −*x*+1, −*y*, −*z*; (ii) −*x*, *y*−1/2, −*z*+1/2.
